# How to Build up a Medical Society

**Published:** 1885-12

**Authors:** 


### "HOW TO BUILD UP A MEDICAL SOCIETY."

THE *Maryland Medical Journal* has an editorial on this subject, and suggests that local societies should follow the example of the Baltimore Academy of Medicine which offers a prize for the best paper read before the Academy during the year; and in addition, recommends that accurate reports of the procedings of a society be made and sent to various journals for publication.

The *Journal of the American Medical Association* indulges in a three column editorial comment on the suggestions, and goes them better by recommending a prize to be offered for the best *discussion* of a paper· We cannot agree with our learned contemporary on this last point; that would be literally "running the thing into the ground." The former suggestions are good, and our own State Association has anticipated our Maryland contemporary in the matter of offering prizes for the best paper. They will be awarded at the coming meeting at Dallas in April.

In the editorial of the last named journal the writer says lugubriously, "Of the forty-three or forty-four hundred regular physicians in Illinois, there are not two thousand who are members of medical societies." We can beat that: Of the forty-three hundred or forty-four hundred physicians in Texas only about four hundred and fifty belong to the State Medical Association—or about ten per cent. This is a lamentable state of affairs which THIS JOURNAL is bending its best energies to correct.

APPROPOS, the *Journal A. M. A.* says:

"Every physician should consider it his duty to belong to at least one medical society; and his second duty is to attend the meetings. Very many physicians seem to regard membership in a medical society as something accidental or incidental to professional life, rather than a duty which one owes to himself and his profession. * * * From one county in Illinois, we learn that 'there is no medical society in this county; the profession is divided.' The divided profession should remember that a society is for the good of the whole, as well as for the benefit of individual members."

That's it, "the profession is divided." It *must be* united, for, never, in our judgment will it be possible to regulate the practice and "suppress quackery" until thorough organization is effected; nor to get the greatest good to be derived from the experience of the members individually, and collectively. Rally! Attend the Dallas meeting next April and let's have a rousing gathering of the clans—a shaking up of dead bones, and a regeneration. In union there's strength and also protection, justice and our rights. Rally !

